# Hierarchical embedding attention for overall survival prediction in lung cancer from unstructured EHRs

**DOI:** 10.1186/s12911-025-02998-6

**Published:** 2025-04-18

**Authors:** Domenico Paolo, Carlo Greco, Alessio Cortellini, Sara Ramella, Paolo Soda, Alessandro Bria, Rosa Sicilia

**Affiliations:** 1 Unit of Computer Systems & Bioinformatics, Department of Engineering, University Campus Bio-Medico di Roma, Roma, Italy; 2 Research Unit of Radiation Oncology, Department of Medicine and Surgery, University Campus Bio-Medico di Roma, Roma, Italy; 3 Operative Research Unit of Radiation Oncology, Fondazione Policlinico Universitario Campus Bio-Medico, Roma, Italy; 4 Operative Research Unit of Medical Oncology, Fondazione Policlinico Universitario Campus Bio-Medico, Roma, Italy; 5 Department of Diagnostics and Intervention, Radiation Physics, Umeå University, Umeå, Sweden; 6 Department of Electrical and Information Engineering, University of Cassino and Southern Latium, Cassino, Italy

**Keywords:** Attention mechanism, Transformer, NER, Unstructured EHRs, Survival analysis, Lung cancer

## Abstract

The automated processing of Electronic Health Records (EHRs) poses a significant challenge due to their unstructured nature, rich in valuable, yet disorganized information. Natural Language Processing (NLP), particularly Named Entity Recognition (NER), has been instrumental in extracting structured information from EHR data. However, existing literature primarly focuses on extracting handcrafted clinical features through NLP and NER methods without delving into their learned representations. In this work, we explore the untapped potential of these representations by considering their contextual richness and entity-specific information. Our proposed methodology extracts representations generated by a transformer-based NER model on EHRs data, combines them using a hierarchical attention mechanism, and employs the obtained enriched representation as input for a clinical prediction model. Specifically, this study addresses Overall Survival (OS) in Non-Small Cell Lung Cancer (NSCLC) using unstructured EHRs data collected from an Italian clinical centre encompassing 838 records from 231 lung cancer patients. Whilst our study is applied on EHRs written in Italian, it serves as use case to prove the effectiveness of extracting and employing high level textual representations that capture relevant information as named entities. Our methodology is interpretable because the hierarchical attention mechanism highlights the information in EHRs that the model considers the most crucial during the decision-making process. We validated this interpretability by measuring the agreement of domain experts on the importance assigned by the hierarchical attention mechanism to EHRs information through a questionnaire. Results demonstrate the effectiveness of our method, showcasing statistically significant improvements over traditional manually extracted clinical features.

## Introduction

The advancement of medicine is inherently tied to the availability and accessibility of extensive clinical data. Among the most valuable sources of information about patient health, Electronic Health Records (EHRs) stand as a vital resource, meticulously documenting the medical history and clinical procedures of individuals.

We can distinguish between structured and unstructured EHRs. Structured EHRs contain organized information, often in a tabular form, which makes the data more comprehensible to computers. In contrast, unstructured EHRs contain information in free text form. EHRs are frequently presented in an unstructured format, posing challenges for systematic processing and analysis [1]. Consequently, automating the processing of data within unstructured EHRs has become a critical challenge in medical research, as it holds the potential to uncover hidden insights and enhance patient care. The use of Natural Language Processing (NLP) tools, specifically Named Entity Recognition (NER) has proven instrumental in extracting meaningful information from these complex medical documents, especially with the introduction of large-scale pre-trained models built on the Transformer architecture [2].

Whilst existing literature has explored methods utilizing NER models to extract information from unstructured data, the embedding representations created by these models have not received due consideration. Transformers have demonstrated an exceptional ability to create dense embeddings of text data capturing the contextual relationships between words and entities. This contextual embedding potentially contains information not only about the presence of individual entities but also related to the relationships and context in which they appear. Effective extraction and use of these enriched informations would offer an opportunity to enhance the performance of automated clinical tasks based of unstructured EHRs analysis.

In this work, we propose HEAL (Hierarchical Embedding Attention for overall survivaL), an interpretable methodology that uses NER-driven EHR representations combined through a hierarchical attention mechanism to highlight the most clinically relevant information in unstructured data for medical applications. As a case of study, we focus on Overall Survival (OS) prediction in Non Small Cell Lung Cancer (NSCLC) patients. NSCLC is the most prevalent form of lung cancer, accounting for an estimated 135,000 deaths per year. Accurate prognosis is crucial for effective treatment planning and improved patient care. Despite the vast amount of clinical data available in unstructured EHRs [3], the narrative sections have not yet been fully utilized for building predictive models in this context. To address this limitation, our model extracts clinically relevant features from EHRs and transforms them into a representation specifically designed to address the unique challenges associated with predicting OS in NSCLC.

Our main contributions are: (i) the development of a completely automated process for extracting a rich representation from unstructured EHR data by utilizing NER and attention mechanisms; (ii) the validation of our approach on a real-world clinical problem, i.e. OS prediction in NSCLC; (iii) the comparison with clinical features manually extracted by human experts, which validates the hypothesis that EHRs can yield more informative features; (iv) the quantitative and qualitative evaluation of attentional maps generated by our model enhancing model transparency and interpretability by domain experts; (v) ablation tests to validate each module of the pipeline, showcasing the importance of a multiclass NER, that refers to the task of identifying and categorizing multiple types of named entities within text.

## Background and motivations

The use of NER has proven instrumental in extracting meaningful information from unstructured EHRs. For many years, research on clinical NER lagged behind the research on general domain NER, mostly due to the lack of available clinical data. To alleviate this problem, research contests (i2b2/n2c2, CCKS, SemEval, etc.) and the research communities (MIMIC, THYME, MEDLINE, etc.) provided public datasets that are highly correlated with the progress of clinical NLP. The application of machine learning and deep learning methods, such as CRF [30] and BiLSTM [31], in clinical NER tasks resulted in remarkable results [32]. However, the actual boost to this field was provided by the advent of large-scale pre-trained models built on the BERT architecture, a deep learning model based on the Transformer paradigm. This is evident in Table 1 which shows the most recent papers in the NER context applied to EHRs. Specifically, we observe that 8 out of 12 employ a BERT-based approach, which aligns with the methodology utilized in our study. Concerning the clinical entities domain, most papers cover general topics and only three papers focus on a specific pathology [22, 26, 29]. This implies a lack of depth and detail on a single disease or condition, which could limit the understanding and practical application of information. In other words, there is not enough focus on specific issues that may require a more in-depth treatment to be understood and managed effectively. Additionally, nearly all of these papers address a multiclass problem, given the prevalence of multiple entities rather than just one. In light of this, our approach involves implementing a multiclass NER system. We believe that the distinctive classes within the NER embeddings can provide significant benefits when using such representations to train a predictive model. Among the literature examined, we found only two papers that include NER to build a predictive model [23, 25], but they do not use NER embeddings as feature representations. Instead, entities are extracted through a NER system and subsequently transformed into numerical representations, primarily using various embedding techniques such as BERT-based models or Word2Vec. This process, however, with a Transformer based model results in the loss of EHRs contextual information surrounding the entities, which can provide valuable insights for a more comprehensive and accurate understanding. In terms of predicting overall survival, which measures the length of time patients remain alive from diagnosis or treatment initiation, conventional studies have predominantly relied on manually extracted clinical features [33, 34]. However, there is a significant gap in research where NER is underutilized for the analysis of unstructured EHRs as a primary data source for prognostic predictions. Integrating NER into survival prediction models offers the potential to uncover previously unrecognized patterns and associations within EHR data, ultimately enhancing the accuracy of prognostic assessments.

**Table 1:** Recent advances in the State-of-the-Art of NER applied to EHR

Model	Ref.	NER Dataset	Application	Entities	NER Performance	Entity Usage
MC-BERT + BiLSTM + CNN + MHA + CRF	[4]	CCKS17 [5], CCKS19 [6], cEHRNER [7]	NER in clinical notes	9 entities: Body, Treatment, Signs, Check, Disease, Lab, Medicine, Operation, Symptom	F1: 94.2%, 86.5%, 92.3% on CCKS17, CCKS19, cEHRNER	None
BiLSTM-CNN-Char	[8]	2010 i2b2/VA [9], 2014 n2c2 [10], 2018 n2c2 [11]	NER in clinical notes	4 entities: Medical Problem, Treatment, Test, Drug	F1: 87.6%, 96.1%, 89.9% on i2b2/VA, 2014 n2c2, 2018 n2c2	None
MUSA-BiLSTM-CRF	[12]	CCKS17 [5], CCKS18 [13]	NERin clinical notes	5 entities: Disease, Symptom, Examination, Treatment, Body part	F1: 92.0%, 91.8% on CCKS17, CCKS18	None
BERT	[14]	2018 n2c2 [11], 2009 n2c2 [15], 2010 n2c2 [9], 2012 n2c2 [16], ShARe13 [17]	NER in clinical notes	4 entities: Drugs, Dosages, Reasons, Adverse drug events	F1: 90.0%, 80.9%, 88.4%, 87.5%, 82.6% on 2018 n2c2, 2009 n2c2, 2010 n2c2, 2012 n2c2, ShARe13	None
BERT-BiLSTM-CRF	[18]	ShARe13 [17], ShARe14 [19]	NER in clinical notes	1 entity: Disorder	F1: 79.9%, 80.7% on ShARe13, ShARe14	None
BERT	[20]	i2b2-2010 [9], VietBioNER [21]	NER in clinical notes	3 entities: Medical Problem, Treatment, Tests	F1: 87.7%, 80.9% on i2b2-2010, VietBioNER	None
CancerBERT	[22]	Proprietary dataset (EHRs)	Breast cancer phenotypes	8 entities: Hormone receptor type, Hormone receptor status, Tumor size, Tumor site, Cancer grade, Histological type, Tumor laterality, Cancer stage	F1: 87.6%	None
scispaCy	[23]	MIMIC-III [24]	NER in clinical notes	2 entities: Disease, Chemical	None	Mortality prediction
med7	[25]	MIMIC-III [24]	NER in clinical notes	7 entities: Dosage, Drug, Duration, Form, Frequency, Route, Strength	None	Mortality prediction
Rule-based	[26]	CCKS20 [27], gastroscopy text dataset, mixed dataset	Breast cancer phenotypes	6 entities: Disease, Anatomy, Imaging, Lab, Drug, Operation	F1: 87.9%, 99.8%, 96.2% on CCKS20, gastroscopy text, mixed dataset	None
Ensemble of CRF, multilingual Transformers (BERT, XLM RoBERTa) and LSTM	[28]	Proprietary dataset (hospital EHRs)	NER in clinical notes	11 entities: Clinical Dept, Date, Duration, Evidential, Frequency, Occurrence, Problem, Test, Time, Treatment, Value	F1: 89.2%	None
RoBERTa	[29]	Proprietary dataset (hospital EHRs)	Breast cancer information	23 entities related to Breast Cancer domain	F1: 95.0%	None

## Methods

**Fig. 1:**
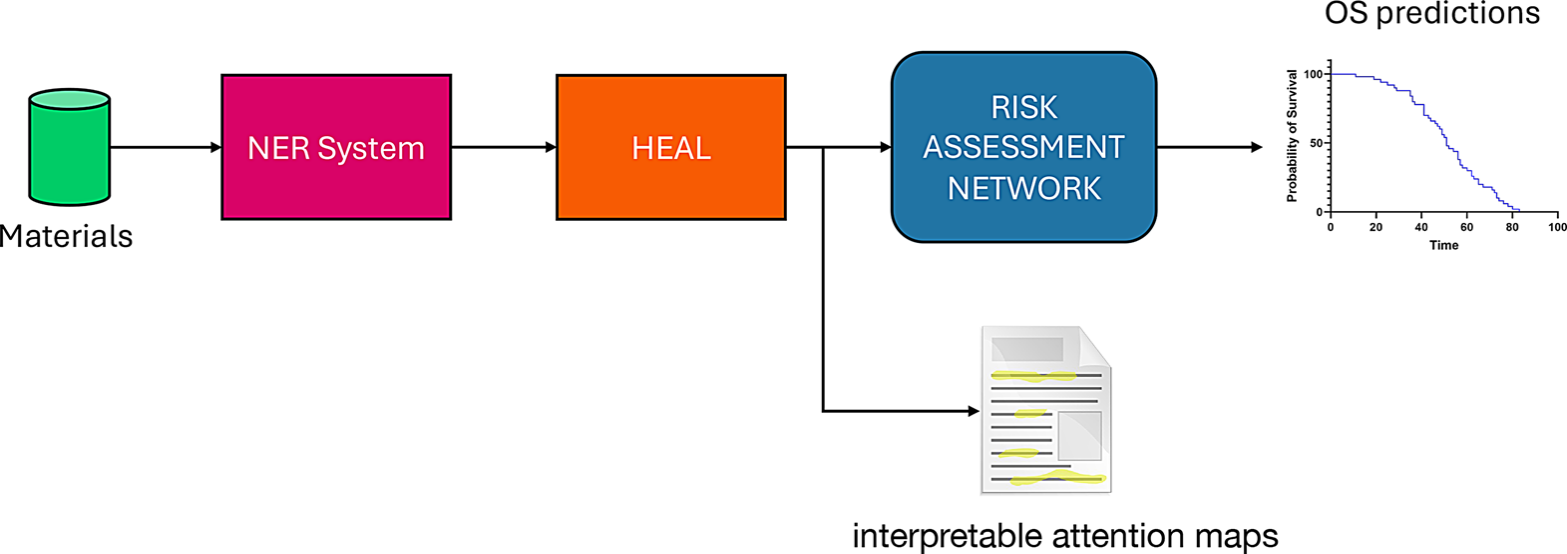
Proposed approach

The proposed approach is depicted in Fig. 1. It starts with a collected dataset of EHRs, which serves as input to a NER system for generating embedding representations of words within each EHR sentence. The subsequent stage involves HEAL, where a hierarchical attentional mechanism is employed for weighted aggregation of embedding representations. Initially, words are aggregated within each sentence, followed by aggregation at the sentence level across patient reports. The resulting output is then forwarded to a risk assessment network. The outputs consist of patients’ OS predictions and associated explanations, reflecting the significance attributed to report sentences by the attention mechanism for the prognostic task. Subsequent sections provide a more detailed examination of these components.

### Materials

We included clinical reports from the CLARO dataset [35, 36], which comprises 231 patients diagnosed with stage III and IV NSCLC. In total, we collected 829 clinical reports, covering reports about oncological and radiotherapy visits. These reports were gathered prior to the initiation of each patient’s therapy and include a comprehensive array of patient information, such as personal data, medical history, reason for visit, notes on histology and imaging, physical examinations, preliminary diagnosis, prescriptions and advice, conclusions, and follow-up details.

The population was enrolled under two different approvals of the Ethical Committee: the first approved on 30 October 30 2012 and registered at ClinicalTrials.gov on 12 July 2018 with Identifier NCT03583723; the second approved on 16 April 2019 with Identifier 16/19 OSS. Written informed consent was obtained from all patients. The authors confirm that all ongoing and related trials for this intervention are registered.

### NER system

NER in EHRs is a NLP technique aimed at the automatic recognition and classification of biomedical entities. These entities can be individual words or phrases within a text that pertain to predefined biomedical categories, referred to as *entity types*. These entity types provide fundamental clinical information with respect to a specific objective, such as diagnosis, patient health status, therapy, etc.

**Fig. 2:**
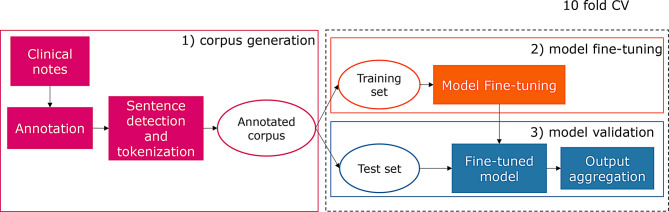
Proposed NER approach. Panel 1) shows the corpus generation, including annotation and the pre-processing of the raw text (sentence detection & tokenization). Panel 2) shows the fine-tuning phase, whereas panel 3) the validation phase. Both 2) and 3) are carried out considering a 10 fold cross-validation experimental setup (10 fold CV black dotted box)

Our proposed NER approach, illustrated in Fig. 2, consists of three steps: corpus generation, model training, and model validation. The first step involves annotating clinical notes, followed by sentence detection and tokenization. With the assistance of two domain experts, we defined 25 entity types related to the NSCLC domain, as detailed in Table 2, and performed the annotation using Doccano [37].

**Table 2:** Entity types acronyms and descriptions, sorted alphabetically based on the “Entity type” column

Entity type	Acronym	Description
Anatomical position	POS	The specific anatomical location of the cancer or anomaly, such as the right lung.
Cancer	CAN	Physicians’ descriptions of tumors (e.g. ‘adenocarcinoma’) and metastasis concepts (e.g. ‘bone metastasis’).
Cancer stage	STA	The stage of the tumor at the time of diagnosis.
Comorbidity	COM	Diseases or conditions that co-occur with a cancer diagnosis.
Date	DAT	Dates of exams, diagnosis, and follow-up implicity mentioned in the clinical notes.
Dosage	DOS	The dosage of drugs (e.g., 25 mg) and therapy.
Drug	DRU	The names of drugs used in the treatment of cancer patients.
Exam	EXA	All the medical examinations undergone by a cancer patient.
Familiarity	FAM	Cancer cases in the patient’s family clinical history.
Focal anomaly	FAN	Any type of abnormality or suspicious pathology, such as nodules and lesions.
Height	HEI	Height of a cancer patient.
Histology	HIS	Histological characteristics of the cancer, such as ‘squamous’.
Mass	MAS	Abnormal growth of cells that forms a mass or tumor within the tissue.
Medication frequency	FRE	The frequency of medication administration.
Morphology	MOR	Shape and structure of the tumor, such as a solid formation with irregular margins.
Numerical Rating Scale	NRS	Pain level on a scale from 0 to 10, with 0 indicating no pain and 10 representing the worst tolerable pain.
Patient event	PEV	When a treatment has been given to a patient, reduced, changed, or discontinued.
Patient symptom	PSY	Symptoms experienced by the patient.
Quantity habits	QHA	The quantity of cigarettes smoked by the patient or alcohol consumed.
Therapy	TPY	The name of the therapy used to treat patients, including radiotherapy and surgery.
Therapy duration	DUR	The duration of a patient’s cancer treatment or the period during which a specific drug was administered.
Teraphy line	TPL	The number of cycles within a therapy.
TNM classification	TNM	T describes the tumor’s size, N indicates the status of nearby lymph nodes, M indicates the presence of metastasis.
Tumor progression	TUP	Changes in the rate of growth or invasiveness of cancer cells.
Weight	WEI	Weight of a cancer patient.

In the annotated corpus, sentences were separated using the dot character (‘.’) and the double new line character (‘\ n\ n’), as two occurrences typically indicated the start of a new sentence. After sentence detection, each sentence underwent tokenization, where it was broken into atomic units using various separators, such as spaces, brackets, and punctuation marks. This process resulted in the creation of a corpus comprising annotated sequences, with an entity type label assigned to each token. The annotated corpus was then iteratively split into training and test sets using a stratified 10-fold cross-validation *per patient*, meaning that reports from the same patient were entirely included in a single fold.

To assess the reliability of the annotations, we compared a second independent clinician’s annotations with the original using the F1-score [38], a measure favored in prior studies [39–41]. The F1-score was computed both at the token and entity levels. At the token level, correct annotations are those with mutual agreement between annotators, while at the entity level, full agreement across all tokens is required for correctness. The average IAA scores were $0.98 \pm 0.04$ for tokens and $0.97 \pm 0.08$ for entities, indicating overall reliability.

**Fig. 3:**
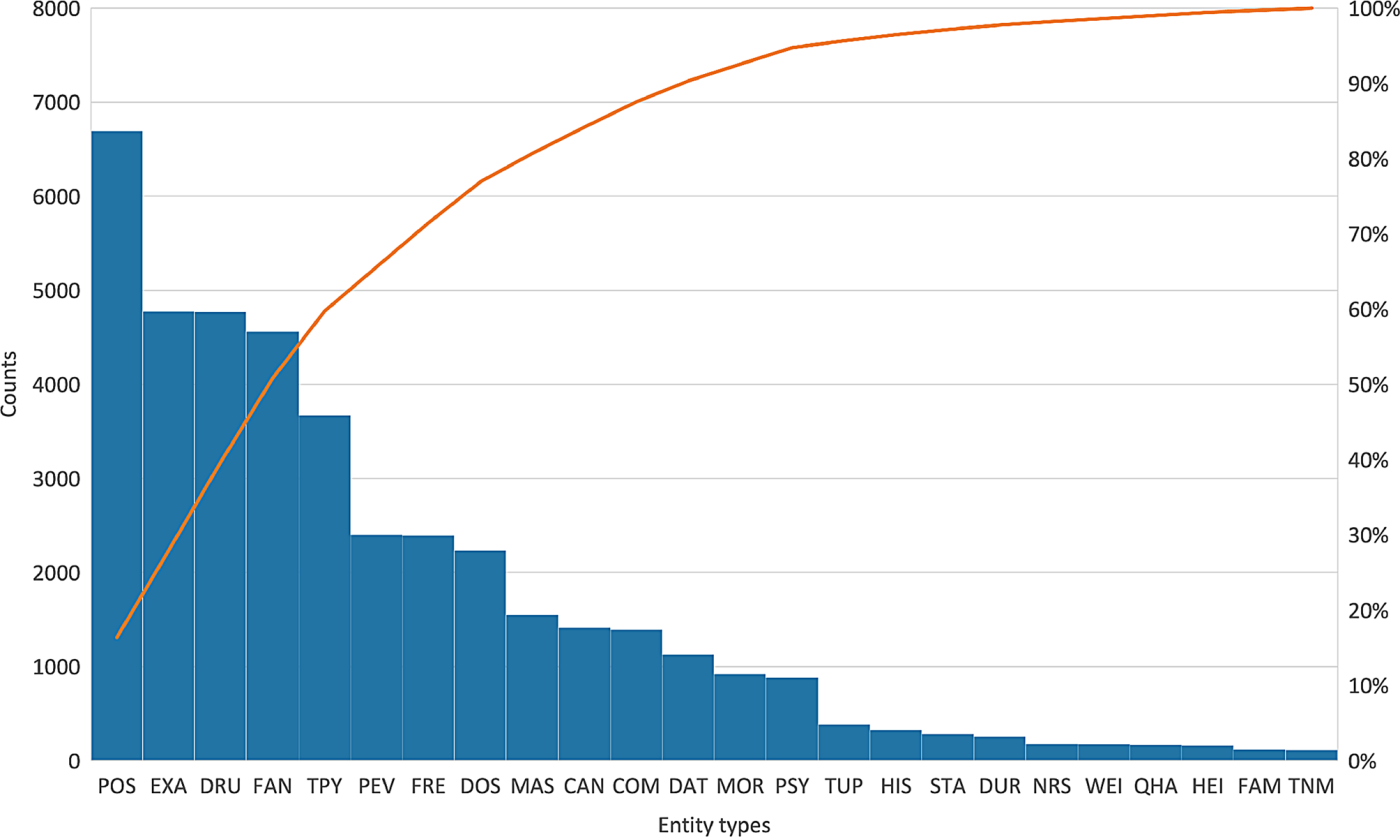
Histogram of entity types. On the y-axis we show the count (on the left) and the a-priori class probability (on the right) of each entity type. On the x-axis we show the various entity types. In addition to the histogram, we also display the Lorenz curve (in orange), which illustrates the distribution of entities in terms of their occurrences

In the second step, we fine-tuned the $$MedBI{T_{R3}} + $$ checkpoint, derived from the pre-trained Biomedical BERT for ITalian (BioBIT) [42], on the training set. BioBIT uses Italian translations of English resources and a domain-specific Italian corpus. We chose $$MedBI{T_{R3}} + $$ for its strong performance in NER tasks [42]. Fine-tuning adapts it to recognize NSCLC-specific biomedical entities, addressing class imbalance in our dataset (Fig. 3), where some entity types (e.g., FAM) are less frequent than others (e.g., POS). To mitigate this, we applied the focal loss function [43], effective in NER tasks [44]. Specifically, we adopted the focal loss function as described in [45].

In the third step, the fine-tuned model was evaluated on the test set using F1-score, Precision, and Recall. Entities were considered correctly predicted only when all tokens matched the ground truth exactly. The performance of $$MedBI{T_{R3}} + $$ was compared with mBERT [46] and UmBERTo [47], demonstrating that $$MedBI{T_{R3}} + $$ outperformed both with an F1 score of $$84.3\% \pm 9.4\%.$$ This result aligns with the scores reported in Table 1, confirming the consistency of the model’s performance with existing research [45]. This success can be attributed to $$MedBI{T_{R3}} + $$’s specialized pre-training on Italian biomedical texts, which allows it to deeply understand medical terminology and nuances, crucial for accurately interpreting clinical reports.

## HEAL: hierarchical embedding attention for overall survival prediction

Before feeding survival data into the risk assessment neural network, a hierarchical attentional mechanism is employed to generate a comprehensive patient representation from all sentences within their clinical reports.

**Fig. 4:**
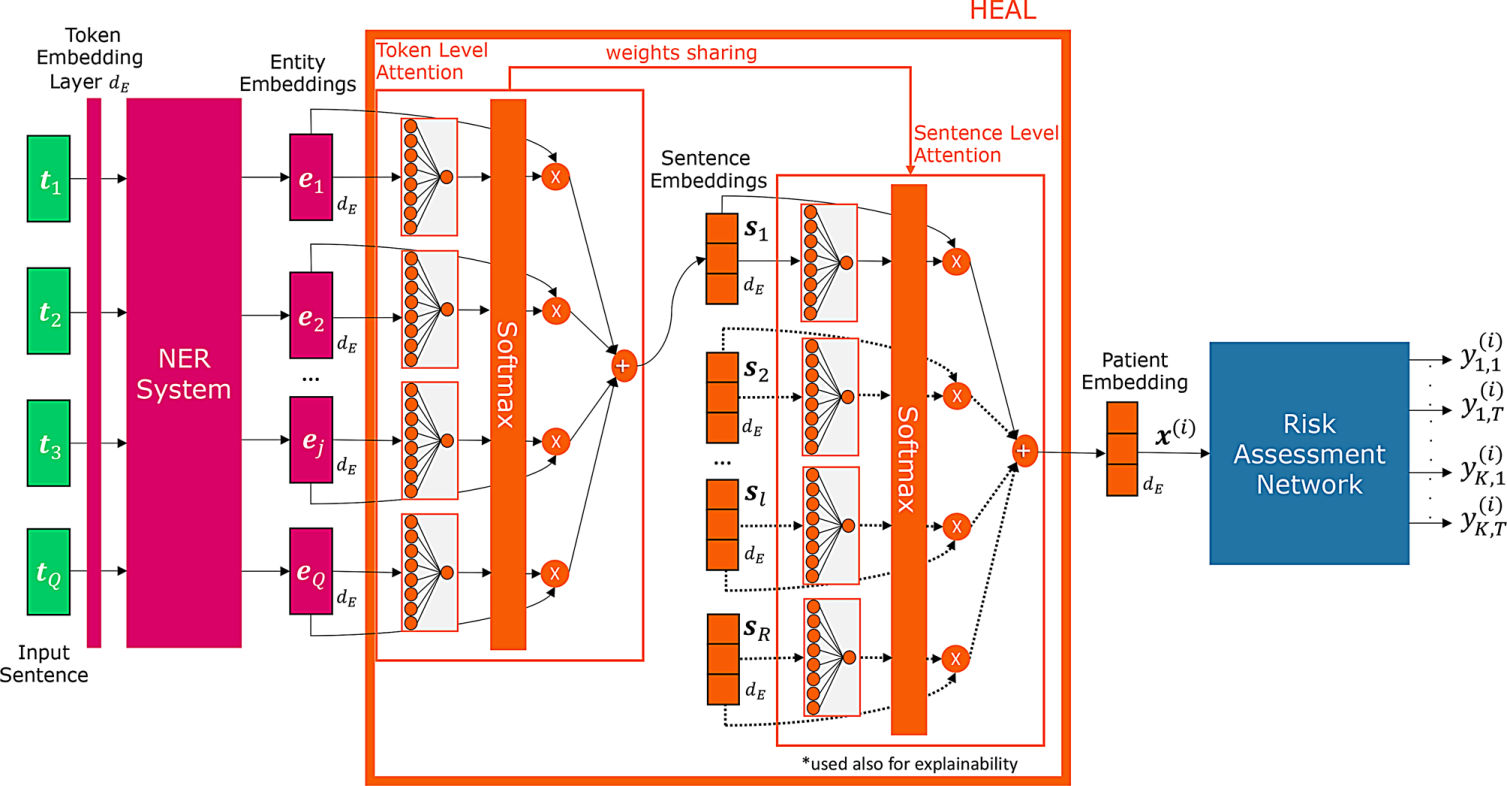
Proposed architecture: The architecture utilizes token embeddings generated by the NER system before the classification layer. Each token embedding classified by the NER system as an entity (Entity Embedding) undergoes a weighting process through a token attentional layer. This produces a weighted average of the same embedding size, named as Sentence Embedding. The sentence embeddings derived from all sentences in a patient’s clinical reports, are then fed into a sentence attentional layer, which shares weights with the token attentional layer. The outcome is a weighted average vector, maintaining the original embedding size ${d_E}$, referred to as the patient embedding ${{\mathbf{x}}^{(i)}}$. The patient embedding is the input of the risk assessment network

HEAL is depicted in Fig. 4 and presented in the following subsections

### Problem statement

Survival data offers three essential pieces of information for each instance or patient: observed features, time elapsed since features were first collected, and a label indicating whether the event (e.g., death) has occurred. In our approach, we consider survival time as discrete, with a finite time horizon. The time set, denoted as $$T,$$ is defined as $$T$$ = {$$0$$, …, $${T_{max}}$$}, where $${T_{max}}$$ represents the predetermined maximum time horizon. Given that the event of interest may not always be observed due to patients being lost to follow-up, survival data often involve censoring. Censoring happens when the observation of an individual ends before the event of interest occurs. Patients may cease participating in the study or being monitored before the event of interest takes place. Addressing this challenge is a crucial aspect of our analysis. We define *censoring* as the event 0 and the set of possible events, including censoring, as $$K = \{ 0,1\} $$, where 1 represents the event of interest, i.e. the death of the patient. Each data point or instance is therefore a triple ($${\mathbf{x}},$$$$s$$, $$k$$) where $${\mathbf{x}}$$$$ \in $$$$X$$ is a $$D$$-dimensional vector of features, $$s$$$$ \in $$$$T$$ is the time at which the event ‘death’ or censoring occurred, and $$k$$$$ \in $$$$K$$ is the event ‘death’ or censoring that occurred at time $$s$$. The dataset $$D = \{ ({{\mathbf{x}}^{(i)}},{s^{(i)}},{k^{(i)}})\} _{i = 1}^N$$ describes a finite set of observed instances or patients in our analysis. For each tuple $$({{\mathbf{x}}^{(i)}},{s^{(i)}},{k^{(i)}})$$ with $${k^{(i)}} \ne 0$$ our focus lies in determining the actual probability $$P(s = {s^{(i)}},k = {k^{(i)}}|{\mathbf{x}} = {{\mathbf{x}}^{(i)}})$$. This probability models the likelihood that a patient with features $${{\mathbf{x}}^{(i)}}$$ will encounter the event $${k^{(i)}}$$ at the specific time $${s^{(i)}}$$. Given the inherent limitation that the true probability cannot be precisely ascertained from any finite dataset, our objective is to derive estimates $$\hat P$$ that serve as approximations to these true probabilities.

### Hierarchical attention mechanism

For each patient the vector of features ${{\mathbf{x}}^{(i)}}$ is derived by harnessing NER-driven representations extracted from their clinical reports and a hierarchical attention mechanism to combine these representations. Specifically, for each token identified as part of an entity within a sentence, we extract the embedding representation ${{\mathbf{e}}_j}$ of size ${d_E}$ generated by the NER system prior to the classification layer as shown in Fig. 4. In this approach, only the embeddings of the entities identified within the sentence are considered, without incorporating the surrounding context. However, the loss of information is mitigated by the contextualized token representations produced by the NER Transformer model, which inherently captures broader contextual information [2]. Additionally, sentences that do not contain any entity tokens are discarded, as they do not provide relevant information for the NER model.

Subsequently, we introduce a weighting step trough a soft attention layer (token attentional layer), that provides the sentence embedding ${{\mathbf{s}}_l}$ as follows:

1 $${{\mathbf{s}}_l} = \sum\limits_{j = 0}^Q {w_j}{{\mathbf{e}}_j}$$

where ${w_j}$ is the weight produced by the soft attention for the token embedding ${{\mathbf{e}}_j}$, $Q$ is the total number of tokens in the input sentence and $l$ ranges from 1 to the total number of sentences $R$ in the patient clinical reports that contain at least one token classified as an entity. Since each sentence may contain a variable number of tokens and each clinical report consists of a different number of sentences, both $Q$ and $R$ are defined dynamically at runtime for each mini-batch. Specifically, $Q$ is set to the maximum number of tokens in a single sentence across all sentences in the mini-batch, while $R$ is defined as the maximum number of sentences containing at least one token classified as an entity across all patients’ clinical reports in the mini-batch. To ensure uniform tensor shapes in the mini-batch, we use padding with all-zero vectors of size ${d_E}$, which receive a weight of zero from both the token-level and sentence-level attention layers, ensuring they do not contribute to the final representation. It is worth nothing that ${{\mathbf{s}}_l}$ has the same size ${d_E}$ of the token embedding.

The sentence embeddings, originating from all sentences within a patient’s clinical reports, are then inputted into a sentence attentional layer, i.e., the right most block in Fig. 4 that compute

2 $${{\mathbf{x}}^{(i)}} = \sum\limits_{l = 0}^R {w_l}{{\mathbf{s}}_l},$$

where ${w_l}$ is the weight produced by the sentence attentional layer for the sentence embedding ${{\mathbf{s}}_l}$. It is a comprehensive representation of the patient’s clinical information and serves as the vector of features for the risk assessment network in Fig. 4. It is worth noting that this layer incorporates a soft attention mechanism that shares weights with the token attentional layer. This is possible because both the token and sentence embeddings have the same dimensionality, ${d_E}$, allowing the use of a single soft attention layer implemented as a fully connected layer of size ${d_E}$. The purpose of weight sharing is twofold: first, to decrease the overall number of network parameters, thereby mitigating overfitting on small data and reducing computational complexity; second, to enhance the transferability of features between layers since the clinical relevance of a sentence is correlated to the clinical relevance of a recognized clinical entity. Hence, the result is a weighted average vector, maintaining the same embedding size ${d_E}$ and named as the patient embedding.

### Risk assessment network

The body of literature addressing survival analysis often approaches the event of interest as the first hitting time of an underlying stochastic process. In a medical context, survival analysis pertains the duration a patient survives. A significant challenge in survival analysis involves understanding the relationship between the distribution of hitting times and the covariates, which represent individual features. Previous research in this field often assumes a specific form for the underlying stochastic process, utilizing available data to learn the relationship between covariates and the parameters of the model, subsequently deducing the connection between covariates and the distribution of first hitting times, also known as the risk of the event (e.g., the risk of death). In this paper we take a markedly different approach to survival analysis by leveraging a deep neural network named DeepHit [33]. DeepHit learns the distribution of first hitting times directly, without making assumptions about the form of the underlying stochastic process. The objective is to instruct the risk assessment network to acquire the knowledge of $\hat P$, the estimate for the joint distribution of the first hitting time and competing events. DeepHit consists of a shared sub-network (SN) and multiple cause-specific sub-networks (CSNs), contingent upon the number of events $k$. To guarantee the learning of the joint distribution of $k$ competing events, as opposed to the marginal distributions of individual events, DeepHit employs a single softmax layer as its output layer. Furthermore, the model incorporates a residual connection linking the input covariates to the input of each cause-specific sub-network, contributing to the overall robustness and effectiveness of the learning process. In our specific context, the sole event under consideration is the patient’s death, denoted as $k = 1$. Consequently, we have just one cause-specific subnetwork (CSN). The shared sub-network and the cause-specific sub-network are composed of ${L_S}$ and ${L_C}$ fully-connected layers, respectively. Here, ${L_S}$ and ${L_C}$ represent the hidden layers of the SN and CSN. The number of these layers, along with the number of hidden neurons, is determined through random search optimization, detailed in subsection 5.3. The shared sub-network takes clinical covariates ${{\mathbf{x}}^{(i)}}$ as inputs and generates an output vector ${f_s}({{\mathbf{x}}^{(i)}})$ capturing the latent representation of covariates. On the other hand, the cause-specific sub-network takes pairs $z = ({f_s}({{\mathbf{x}}^{(i)}}),{{\mathbf{x}}^{(i)}})$ as inputs and produces an output vector ${f_c}(z)$ representing the probability of the first hitting time. Notably, these sub-networks incorporate both the output of the shared network and the original covariates as inputs. This design choice allows the sub-networks to access the learned common representation ${f_s}({{\mathbf{x}}^{(i)}})$ while retaining the ability to learn distinct aspects of the representation. The softmax layer generates a probability distribution denoted as ${{\mathbf{y}}^{{\mathbf{(i)}}}} = [y_1^{(i)},\ldots,y_{{T_{max}}}^{(i)}]$, where $y_s^{(i)}$ represents the estimated probability $\hat P({s^{(i)}},{k^{(i)}}|{{\mathbf{x}}^{(i)}})$ indicating the likelihood of the patient $i$ experiencing event ${k^{(i)}} = 1$ at the time ${s^{(i)}}$. This architectural framework encourages the network to grasp potentially non-linear and even non-proportional relationships between covariates and associated risks. To assess the risk of event occurrence, the cause-specific cumulative incidence function (CIF), expressed as ${F_{{k^{(i)}}}}({s^{(i)}}|{{\mathbf{x}}^{(i)}}),$ is employed. This function quantifies the probability of the event ${k^{(i)}} = 1$ occurring on or before time ${t^{(i)}}$, given the covariates ${{\mathbf{x}}^{(i)}}$. Formally, the CIF for event ${k^{(i)}} = 1$ is expressed as:

3 $${F_{{k^{(i)}}}}({t^{(i)}}|{{\mathbf{x}}^{(i)}}) = \sum\limits_{{s^{(i)}} = 0}^{{t^{(i)}}} P(s = {s^{(i)}},k = {k^{(i)}}|{\mathbf{x}} = {{\mathbf{x}}^{(i)}}).$$

However, since the true CIF ${F_{{k^{(i)}}}}({s^{(i)}}|{{\mathbf{x}}^{(i)}})$ is not known, we utilize the estimated CIF

4 $${\hat F_{{k^{(i)}}}}({t^{(i)}}|{{\mathbf{x}}^{(i)}}) = \sum\limits_{m = 0}^{{s^{(i)}}} {y_{1,m}}.$$

### Loss function

In our implementation of DeepHit we define a loss function $\mathcal{L}$, that has been specifically crafted to effectively handle censoring data. It is expressed as the formula $\mathcal{L} = \alpha {\mathcal{L}_1} + \beta {\mathcal{L}_2} + \gamma {\mathcal{L}_3}$ where $\alpha$, $\beta$, $\gamma$ weight the three terms ${\mathcal{L}_1}$, ${\mathcal{L}_2}$, ${\mathcal{L}_3}$ now described. The term ${\mathcal{L}_1}$ embodies the log-likelihood of the joint distribution concerning the first hitting time and the unique event, i.e. death (${k^{(i)}}$=1). Notably, this formulation has been adapted to accommodate the presence of censored data. For patients who have not experienced censoring, ${\mathcal{L}_1}$ encapsulates both the occurrence of the event and the corresponding time at which it occurred. On the other hand, for patients who have been subject to censoring, ${\mathcal{L}_1}$ effectively captures the time at which the patient becomes censored, indicating that they were alive up to that specific point in time and providing valuable information regarding their status at that juncture. This adjustment ensures that censoring is appropriately accounted for, offering a more accurate representation of patient outcomes. Formally

5 $$\begin{array}{*{20}{l}} {{\mathcal{L}_1} = - \sum\limits_{i = 1}^N [{1}({k^{(i)}} \ne 0) \cdot log(y_{{k^{(i)}},{s^{(i)}}}^{(i)})} \\ { + {1}({k^{(i)}} = 0) \cdot log(1 - \sum\limits_{k = 1}^K {{\hat F}_k}({s^{(i)}}|{{\mathbf{x}}^{(i)}}))],} \end{array}$$

where ${1}()$ is an indicator function and $N$ is the number of patients in the dataset. The first term captures the information contributed by patients who have not undergone censoring. The second term addresses censoring bias by leveraging the understanding that these patients are confirmed to be alive at the time of censoring. This acknowledgment enables the model to anticipate that the first hitting event will occur after the specified censoring time.

${\mathcal{L}_2}$ integrates a blend of cause-specific ranking loss functions, and it uses the estimated CIFs computed at various times, corresponding to the instances when events actually occur. This approach is employed to fine-tune the network for each cause-specific estimated CIF. Since this study focuses on a single event, there is only one cause-specific estimated CIF. Our methodology employes a ranking loss function that incorporates the concept of concordance: a patient experiencing an event at time $s$ should exhibit a higher risk at that specific time $s$ than a patient who has survived beyond $s$. This ensures that the model not only predicts the occurrence of the event, but also correctly orders the risks of death over time. Formally

6 $${\mathcal{L}_2} = \sum\limits_{k = 1}^K {\theta _k} \cdot \sum\limits_{\substack{ i = 1 \\ i \ne j } }^N {A_{k,i,j}} \cdot \eta ({\hat F_k}({s^{(i)}}|{{\mathbf{x}}^{(i)}}),{\hat F_k}({s^{(i)}}|{{\mathbf{x}}^{(j)}})),$$

where the coefficients ${\theta _k}$ are chosen to trade off ranking losses of the $k - th$ competing event, $\eta (a,b)$ is a convex loss function defined as $\eta (a,b) = \exp \left( { - \frac{{(a - b)}}{\sigma }} \right)$ with $\sigma$ set equal to 0.1 and ${A_{k,i,j}}$ is defined as follows:

7 $${A_{k,i,j}} = {1}({k^{(i)}} = k,{s^{(i)}} < {s^{(j)}}),$$

and represents pairs $(i,j)$ acceptable for event $k$. Since this study focuses on a single event, only one coefficient, i.e. ${\theta _1}$, is included, and its value is fixed at 1. The inclusion of ${\mathcal{L}_2}$ in the overall loss function penalizes the misordering of pairs concerning each event. Consequently, minimizing the total loss serves to incentivize the correct ordering of pairs for each event.

${\mathcal{L}_3}$ is a calibration loss: it focuses on how well predicted probabilities align with observed outcomes, ensuring that the model’s predicted risk accurately reflects the true event occurrence. It is defined as follows:

8 $${\mathcal{L}_3} = \sum\limits_{k = 1}^K \frac{1}{N} \cdot \sum\limits_{i = 1}^N ({\hat F_k}({s^{(i)}}|{{\mathbf{x}}^{(i)}}) - {I_i}),$$

where ${I_i}$ represents the indicator of the event, specifically, the death of the patient. When ${I_i}$ equals 1, it signifies the occurrence of the patient’s death. Conversely, when ${I_i}$ equals 0, it indicates truncation.

## Experiments

This section elucidates the comparisons and ablation studies conducted to evaluate the effectiveness and quality of our approach. Additionally, it elaborates on the experimental setting and the performance metric employed for these evaluations.

### Comparison with clinical features

The effectiveness of our method was assessed through a comprehensive comparison with the clinically relevant features manually extracted by human experts listed in Table 3. These features were selected based on the guidance of two domain experts, an oncologist and a radiation oncologist, and had been utilized in previous research [48]. They served as the sole input for the risk assessment network.

**Table 3:** Patients’features

Feature	Description	Values
Gender	The gender of the patient	[M, F]
Overall Stage	The cancer stage	[II, III, IV]
cT	The clinical tumor size classification	[1, 2, 3, 4]
cN	The lymph node classifications	[0, 1, 2, 3]
cM	The metastasis classification	[0,1]
Histology	The specific diagnosis related to the cancer type	[Adenocarcinoma, Squamous, Other, Unknown]
CTV	Clinical Target Volume of tumor	[1.8–568.61] $c{m^3}$
Age	The age of the patient	[29–92]

### Ablation tests

To assess the significance of each module in our proposed architecture, we conducted the following ablation tests:

1. **No NER:** this approach involves combining at first attention layer (token level attention in Fig. 4) the embedding of all tokens within a sentence, not only of those belonging to an entity type, ignoring the NER output. This ensures that all sentences in the patient’s clinical reports are taken into account. It serves to assess the importance of incorporating NER classification output to understand the contribution of NER classification in order to extract discriminative features for OS prediction.
2. **Binary NER (Bi-NER):** in binary NER, a token within a sentence is classified as belonging to an entity or not, without additional subcategorization into specific types defined in Table 2. It serves to evaluate the relevance of multiclass NER as opposed to binary NER in order to extract discriminative features for OS prediction.
3. **No HEAL:** this approach involves substituting HEAL with a simple average among all the entity token embeddings that are outputted by the NER system, without any distinction in sentences. It allows to assess the importance of weighting information in clinical reports.
4. **Only Sentence Attention (SA):** this approach substitutes only the first attention layer (token level attention in Fig. 4) with a simple average among all entity token embedding of a sentence. The sentence level attention is maintained, allowing to understand the importance of weighting sentences within patient clinical reports.
5. **Only Token Attention (TA):** this approach substitutes the second attention layer (sentence level attention in Fig. 4) with a simple average among all sentence token embedding in the patient clinical reports. The token level attention is maintained, allowing to understand the importance of weighting tokens within sentences.
6. **No weight sharing (No WS):** this method specifically eliminates weight sharing between the token level attention layer and the sentence level attention layer. This aspect is crucial for understanding how weight distribution influences the model’s ability to capture complex relationships within the data.
7. **Degradated NER (Deg-NER):** this method utilizes a degraded NER system with an F1 score of 65%, achieved by introducing noise through the random removal of correctly identified entities. This approach allows for the evaluation of how a reduction in entity quality impacts overall model performance.

### Experimental setting

During the training and evaluation of the risk assessment network, a 10-fold cross-validation was implemented on a per-patient basis. Within each cross-validation iteration, an additional stratified inner 10-fold cross-validation was conducted to fine-tune the network hyperparameters reported in Table 4. This optimization process involved a random search with 100 iterations over the hyperparameter space depicted in Table 4. The entire process was repeated five times for each setting (HEAL, clinical features, ablation tests) to address fluctuations in the results, ultimately providing a more reliable and precise perspective on the performance of the risk assessment network. All experiments were implemented in PyTorch and run on a NVIDIA A100 GPU with 40 GB of VRAM. Table 5 shows the durations required for random search, training, and testing within a single fold are depicted for each modality, providing a detailed breakdown of the computational times associated with the experiment. It’s crucial to note that approaches incorporating at least one attention-level layer exhibit higher computational times for random search and training compared to others, particularly evident in scenarios featuring two attention-level layers, as exemplified by HEAL. However, the time required for testing remains comparable to alternative approaches.

**Table 4:** Hyperparameters search space of the risk assessment network

Hyperparameter	Search space
Batch size	[8, 16]
# hidden layers for both SN (${L_S}$) and CSNs (${L_C}$)	[1, 2, 3, 5]
# neurons per hidden layer	[20, 50, 100, 200]
Dropout rate	[0.2, 0.3, 0.4]
Activation function	[ReLU, SELU]
$\alpha$	[0.1, 0.5, 1.0, 3.0]
Loss function $$\mathcal{L}$$$$\beta $$	[0.1, 0.5, 1.0, 3.0]
$\gamma$	[0.1, 0.5, 1.0, 3.0]

**Table 5:** Random search, training and testing times across a single fold

Approach	Random search Time[h]	Training Time[s]	Testing Time[s]	Testing Time per Patient[s]
Clinical features	0.88	3.18	0.10	0.004
No NER	0.73	2.51	0.10	0.004
Bi-NER	52.39	137.80	6.18	0.281
No HEAL	0.75	2.90	0.10	0.004
SA	2.74	11.60	0.39	0.018
TA	20.83	128.1	2.10	0.160
No WS	58.61	140.00	6.18	0.281
Deg-NER	52.39	137.80	6.18	0.281
**HEAL**	52.39	137.80	6.18	0.281

### Performance metric

We use the time-dependent concordance index (${C^{td}}$-index) as our metric of performance, which ranges from 0 to 1. It is important to highlight that the conventional concordance index ($C$-index) [50] is a widely utilized discriminative metric. The C-index operates under the assumption that patients with longer lifespans should be associated with a lower risk compared to those with shorter lifespans. However, the ordinary C-index is calculated solely at the initial observation time, lacking the capacity to capture potential variations in risk over time. In contrast, the time-dependent concordance index considers the temporal aspect, offering a more comprehensive understanding of how risk evolves over the course of observation. The ${C^{td}}$-index for event $k$ is defined as:

9 $$\begin{array}{*{20}{l}} {{C^{td}} = P({{\hat F}_k}({s^{(i)}}|{x^{(i)}}) > {{\hat F}_k}({s^{(i)}}|{x^{(j)}})|{s^{(i)}} < {s^{(j)}})} \\ { \approx \frac{{\sum\limits_{i \ne j} {A_{k,i,j}} \cdot {1}({{\hat F}_k}({s^{(i)}}|{x^{(i)}}) > {{\hat F}_k}({s^{(i)}}|{x^{(j)}}))}}{{\sum\limits_{i \ne j} {A_{k,i,j}}}}.} \end{array}$$

Thus, the ${C^{td}}$-index for event $k$ is computed by comparing pairs of observations. In each pair, one patient has experienced event $k$ at a specific time, whilst the other has neither encountered the event nor been truncated to that time. The significance of this discriminative index lies in its independence from a single fixed time. This characteristic renders it well-suited for situations where the impact of covariates on survival undergoes variations over time. In other words, this index is particularly valuable when risks exhibit non-proportional behavior over the course of observation.

## Results and discussions

Table 6 summarizes the results averaged over 5 runs, presenting the performance metrics in terms of the ${C^{td}}$-index for the compared modality. For the HEAL modality, the model achieved an average ${C^{td}}$-index of 0.639 with a low standard deviation of 0.014, which is lower than the standard deviations of other methods, indicating higher consistency across the runs. Conversely, the model’s performance decreased in No HEAL modality, yielding an average ${C^{td}}$-index of 0.558. This observation highlights the importance of appropriately weighing information within clinical reports for optimal predictive outcomes. The ablation test with only the Sentence Attention (SA) mechanism led to improved performance, with an average ${C^{td}}$-index of 0.624. Although slightly lower than HEAL, this enhancement suggests that strategically weighting sentences inside clinical reports had a positive impact to overall performance. In the absence of weight sharing inside HEAL, the model exhibited an average ${C^{td}}$-index of 0.615, which is slightly lower than HEAL by 0.009. This suggests that the network performs better with a reduced number of parameters, possibly due to training with a limited number of samples. Both the binary NER (Bi-NER) and the no NER modalities resulted in significatively lower performances, with an average ${C^{td}}$-index of 0.546. This indicates the fundamental role of NER label information in training an effective predictive model, emphasizing that tokens not associated with entities are of low informational value. Compared to the binary NER (Bi-NER) and no NER modalities, the degraded NER (Deg-NER) modality demonstrated improved performance, though it remained significantly below HEAL. This highlights not only the essential role of NER label information in predictive modeling but also the necessity of a high-quality NER system to achieve optimal performance. Clinical features exhibited a slightly lower performance with an average ${C^{td}}$-index of 0.590. This suggests that the proposed automated process outperforms manually extracted features by humans. In summary, these results offer insights into the relative effectiveness of different modalities and model configurations in predicting risk, with the HEAL modality emerging as the most consistent and effective among the tested approaches.

**Table 6:** Results of ${C^{td}}$-index for individual modalities obtained from 5 iterations

Approach	${C^{td}}$**-index (mean**$\pm$**std)**
Clinical features	$0.590 \pm 0.019$
No NER	$0.546 \pm 0.029$
Bi-NER	$0.499 \pm 0.023$
No HEAL	$0.558 \pm 0.023$
SA	$0.624 \pm 0.027$
TA	$0.570 \pm 0.037$
No WS	$0.615 \pm 0.033$
Deg-NER	$0.563 \pm 0.026$
**HEAL**	**0.639 ± 0.014**

In order to further validate the difference between the proposed approach and the compared methods, we performed the Student’s t-test in a pairwise fashion, considering HEAL angaist each competitor. The results are summarized in Table 7. A significance threshold $\hat \alpha$ of 0.05 was established for the conducted tests. The results of t-tests again highlight the pivotal role of attention mechanisms:for all competitors lacking attention mechanisms, the p-values consistently fell below the established threshold of 0.05, indicating statistical significance. The significance of the p-value persists even for the TA competitor, where an attention mechanism is present. However, this mechanism aggregates all words in patient clinical reports, disregarding sentence splitting and thus the hierarchical structure of our methodology. Noteworthy are the elevated p-values associated with the other scenarios featuring at least one attention mechanism, specifically in the exclusive presence of the Sentence Attention ($p$-value = 0.290) and the absence of weight sharing ($p$-value = 0.174). Even if the two approaches are similar to HEAL, the ${C^{td}}$-index mean and standard deviation values reveal a consistent trend towards superior and more robust outcomes with HEAL. Attributing our model’s superiority, we highlight two key factors: the inclusion of weighting tokens within sentences before weighting the sentences themselves, and the use of weight sharing. The first significantly enhances the comprehension of clinical information, particularly when dealing with larger data volumes compared to the Sentence Attention (SA) approach. The second reduces the number of parameters compared to the No Weight Sharing (No WS) approach, contributing to the model’s efficiency and effectiveness.

**Table 7:** Statistical analysis of performance differences between HEAL and the other modalities. Statistically significantly differences ($p$-value < 0.05) are highlighted in bold

Approach	Compared to	$$\Delta $$$${C^{td}}$$**-index (mean)**	p-value
**HEAL**	Clinical features	0.049	**0.001**
	No NER	0.093	<**0.001**
	Bi-NER	0.140	<**0.001**
	No HEAL	0.081	<**0.001**
	SA	0.015	0.290
	TA	0.069	**0.004**
	No WS	0.024	0.174
	Deg-NER	0.076	<**0.001**

## Interpretability

**Fig. 5:**
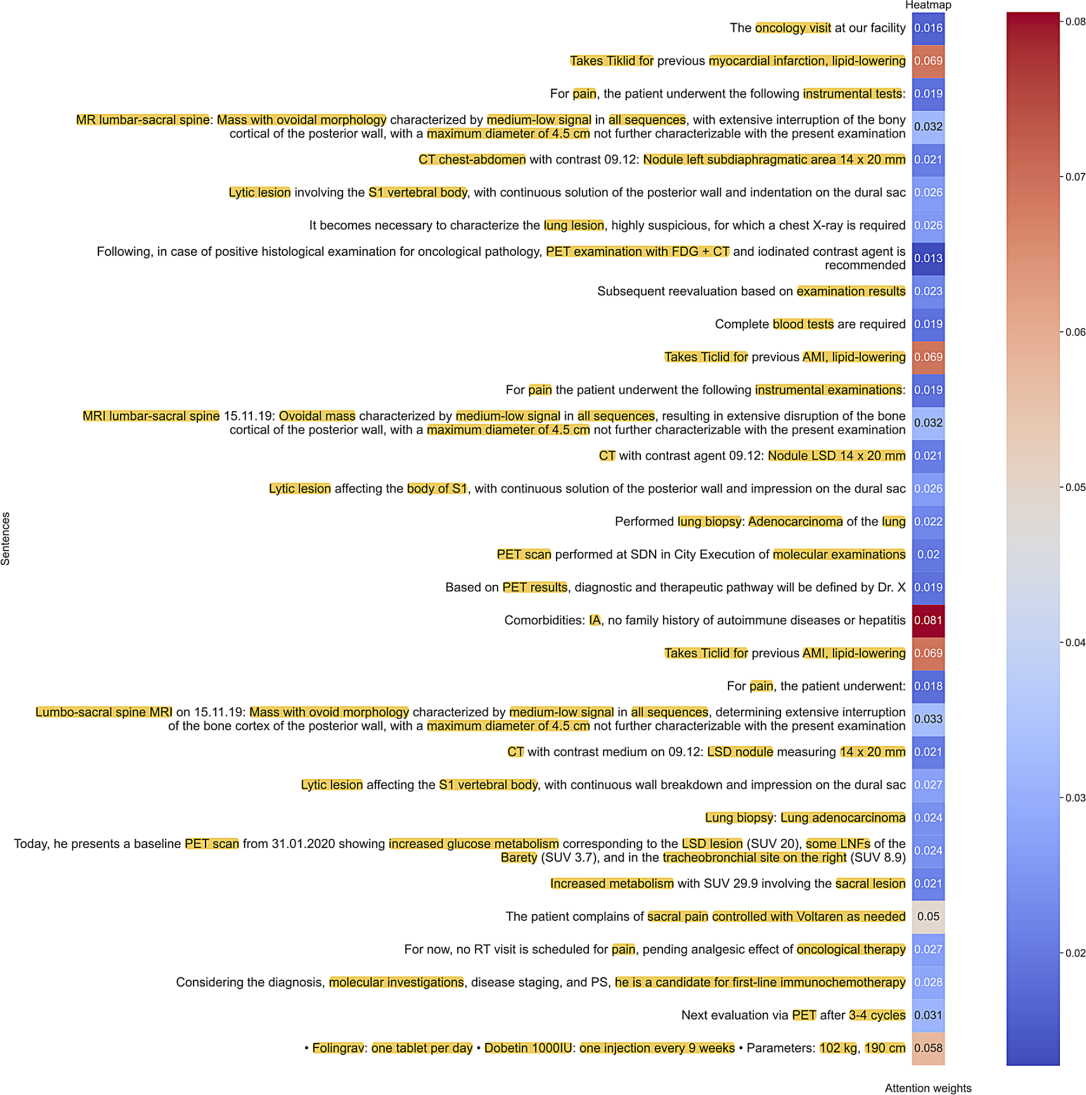
Example of Attentional Map: within each sentence in patient clinical reports, only the words identified as entities by the NER system, highlighted in yellow, are aggregated into the sentence embedding. These sentences receive a score assigned by the second attention layer (sentence level attention), with higher scores depicted in shades of red and lower scores tending towards blue. The text was translated from Italian to English for presentation purposes

**Fig. 6:**
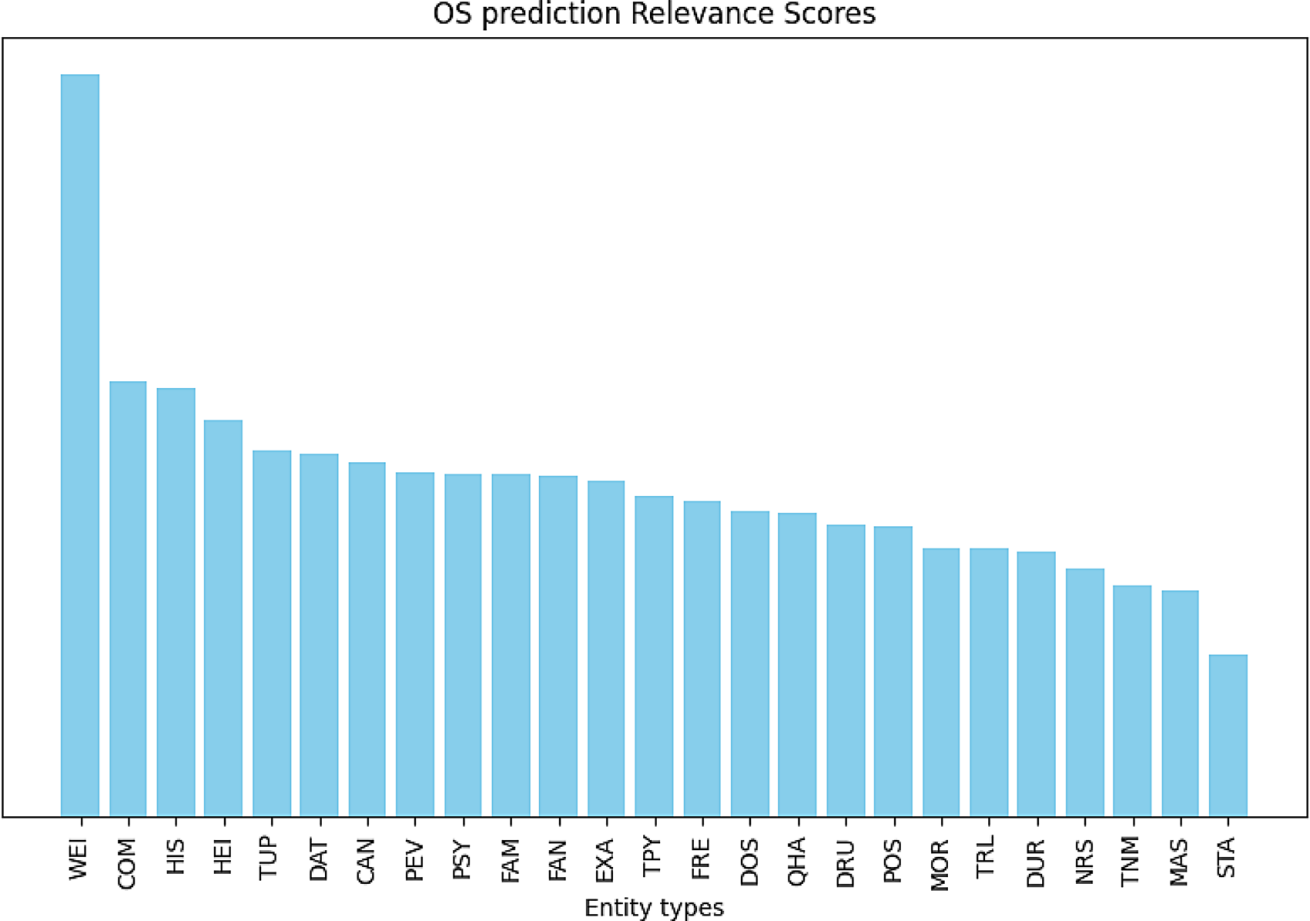
Entity types relevance scores for OS prediction

In deep learning models, the challenge of interpretability arises from the intricate nature of understanding and elucidating the rationale behind model’s specific decisions. In our work, we address this interpretability challenge by leveraging attentional maps generated through the hierarchical attention mechanism in order to highlight the specific portions (sentences) of the input data (clinical reports) that the model deemed most crucial during the decision-making process. An example of attentional map is shown in Fig. 5, which presents sentences extracted from the clinical reports of a patient who has been assigned an 86% risk score of experiencing “death” within 29 months. This score is primarily attributed to the patient’s comorbidities, such as IA (Aortic Insufficiency) and AMI (Acute Myocardial Infarction), as well as treatments like Tiklid and Folingrav, since sentences mentioning these factors received higher scores from the sentence-level attention mechanism (0.081 and 0.069). Interestingly, not only clinical concepts but also attributes like weight and height appear in sentences with high attention scores. Conversely, the names of the exams (PET scans) received less importance, likely because they are routine and not discriminative for the outcome prognosis. This trend is further depicted in Fig. 6, which illustrates the contribution of the different entity types to OS prediction. For each entity type, a relevance score is computed by first averaging the token-level attention scores assigned to each entity occurrence (since an entity may span multiple tokens). The total attention score is then obtained by summing these mean scores across all occurrences of that entity type and dividing it by the number of occurrences, ensuring a fair comparison across entity types. As a result, it is possible to observe that entity types with strong clinical relevance, such as comorbidities and histology, are among the most influential. However, non-clinical factors like weight and height also contribute significantly to the attention scores, highlighting the model’s ability to capture diverse predictive signals.

### Sanity check

To evaluate the robustness and reliability of the model in generating attentional maps we implemented a sanity check that involved comparing attentional maps obtained from a faulty system angaist those from the accurate system. The attentional maps from the faulty system are generated by training the model using randomly permuted OS labels, following a data randomization test [49]. The primary objective of the sanity check is to discern whether the model can distinguish between the true signals influencing its decisions and mere noise or random associations. Cosine similarity analysis between attentional maps of the two systems yielded a mean score of 0.578 with a standard deviation of 0.138, indicating a relatively substantial difference between attentional maps from the faulty and accurate systems.

### Experts agreement assessment

To qualitatively assess the attentional maps generated by our model during the decision-making process, we measures the agreement with domain experts on the importance given by the model to the sentences within patients’ clinical reports for predicting clinical outcomes.

To this end we set up a questionnaire consisting of four questions, each corresponding to an individual patient’s clinical report under examination. For each question, participants assess the level of agreement with the significance assigned by the model to the sentences within the report. In particular, each sentence in the report was highlighted with a specific color denoting its importance. We employed a three-level highlighting system: orange for the most important sentences (those receiving the highest attentional weights from the model), blue for the least important sentences (those receiving the lowest attentional weights from the model) and green for sentences falling in between. For each question, participants have the option to choose from five different response levels: completely agree, agree, neutral, disagree, and completely disagree. These responses were systematically encoded on a numerical scale using the Likert scale, with values ranging from 0 for “completely disagree” to 4 for “completely agree”. The questionnaire was proposed to four domain-experts. We obtained an overall agreement of 67.2%, which suggests a substantial level of consensus among the respondents. This indicates a noteworthy degree of alignment in perceptions regarding the model’s attention to critical information within clinical reports. However, it is important to further explore the remaining 32.8%, where participants have divergent views. Specifically, these divergent perspectives manifest a clinicians’ tendency to attribute higher significance to particular sections in the descriptions of CT and MRI exams, as well as to the diagnosis itself, even though the model does not categorize these details as the least crucial in the reports. Moreover, since the diagnosis is consistent for almost all patients in the cohort, the model may not emphasize this feature significantly when distinguishing between patients for prognostic purposes.

## Conclusions

This paper introduces a novel and interpretable methodology designed to enhance the extraction of clinically significant information from unstructured data. We accomplish this by employing a multiclass NER approach, coupled with a hierarchical attention mechanism. The synergy of these components enables us to highlight the most pertinent clinical details, thereby increasing the relevance of the data, especially in the context of medical applications. We apply our methodology in the context of NSCLC to predict OS. The results of our experiments underscore the significance of employing multiclass NER and the hierarchical attention mechanism in accurately predicting OS in NSCLC. Notably, our findings reveal that the automated system generated by this methodology yields more informative features compared to features manually extracted by human experts.

Beyond the achievements highlighted in this study, our methodology can be tailored to tackle a broader spectrum of clinical prediction tasks, extending beyond the specific focus on overall survival in the context of lung cancer, opening up the possibility of its application in diverse medical domains. For instance, it could be applied to predict disease progression, treatment response, or patient prognosis across various medical conditions beside lung cancer. Moreover, we aspire to create a robust multimodal framework, tailoring the methodology to comprehensively handle diverse modalities beyond the narrative section of EHRs, including images and the structured section of EHRs.

In conclusion, the presented methodology not only advances the understanding of NSCLC prognosis but also lays the foundation for a broader spectrum of clinical prediction applications. Its adaptability, along with the potential to synergize with different data sources, makes it a promising tool for the future of medical research and healthcare.

## Data Availability

No datasets were generated or analysed during the current study.
